# Preoperative Prediction of Intraoperative Transfusion in Pediatric Craniosynostosis Surgery: An Exploratory Prediction Model Study

**DOI:** 10.3390/medicina62050865

**Published:** 2026-04-30

**Authors:** Sung-Hye Byun, Jihyun Woo, Jung A Lim, Sou-Hyun Lee

**Affiliations:** 1 Department of Anesthesiology and Pain Medicine, School of Medicine, Kyungpook National University, Kyungpook National University Chilgok Hospital, Daegu 41404, Republic of Korea; shbyun82@knu.ac.kr; 2 Department of Anesthesiology and Pain Medicine, School of Medicine, Kyungpook National University, Kyungpook National University Hospital, Daegu 41944, Republic of Koreajalim@knu.ac.kr (J.A.L.)

**Keywords:** craniosynostoses, prediction methods, machine, risk assessment

## Abstract

*Background and Objectives:* Craniosynostosis repair is associated with a high perioperative transfusion rate, but preoperative prediction models remain limited. This exploratory study aimed to develop and internally validate clinically prespecified preoperative models for predicting intraoperative red blood cell transfusion in pediatric craniosynostosis surgery and to evaluate whether adding fused suture extent improved model performance. *Materials and Methods*: This retrospective single-center prediction model study included children who underwent craniosynostosis repair between 2014 and February 2026. Patients undergoing repeat procedures or concurrent surgery for other craniofacial anomalies were excluded. The outcome was any intraoperative red blood cell transfusion. Candidate predictors were prespecified as age, weight, American Society of Anesthesiologists Physical Status (ASA-PS), preoperative hemoglobin, preoperative platelet, and fused suture extent. Five paired baseline/full ridge-penalized logistic regression models were developed, with fused suture extent added only to the full models. Performance was evaluated using apparent and bootstrap optimism-corrected area under the receiver operating characteristic curve (AUC) and Brier score. *Results:* Twenty-one patients were included, and nine (42.9%) received intraoperative transfusion. Across all five comparisons, inclusion of fused suture extent improved optimism-corrected discrimination and reduced prediction error. Corrected AUC increased from 0.470 to 0.674, from 0.475 to 0.738, from 0.552 to 0.667, from 0.516 to 0.704, and from 0.466 to 0.694 across the five model pairs. The best-performing model included weight, preoperative hemoglobin, ASA-PS, and fused suture extent, with an optimism-corrected AUC of 0.738 and an optimism-corrected Brier score of 0.242. *Conclusions*: Inclusion of fused suture extent improved preoperative prediction of intraoperative transfusion and may support perioperative blood management planning in pediatric craniosynostosis surgery. However, external validation using larger independent cohorts is necessary prior to clinical implementation.

## 1. Introduction

Craniosynostosis is defined as the premature fusion of one or more cranial sutures. Although it is relatively uncommon, with a reported prevalence of 5.2 per 10,000 live births [1], surgical repair is associated with a high perioperative transfusion rate (34.8–87%) [2,3]. This rate remains markedly higher than the overall perioperative transfusion rate reported for pediatric surgical patients (6.3%) [4]. In addition, one study suggested that the prevalence of craniosynostosis has increased over time [5].

Intraoperative hypotension and anemia have been associated with acute kidney injury and mortality [6,7]. Although several studies have examined factors associated with transfusion during craniosynostosis repair [8,9,10], they have largely focused on intraoperative variables (e.g., anesthesia and operative duration). In contrast, the fused suture site, an important preoperative determinant closely related to surgical extent and anticipated blood loss, has not been specifically evaluated in prior transfusion-risk analyses. The involvement of multiple sutures has been associated with longer and more complex surgical procedures, as well as a higher likelihood of blood transfusion, compared with single-suture craniosynostosis [11,12]

From an anesthesiologist’s perspective, identifying preoperative predictors of intraoperative transfusion is essential to anticipate substantial blood loss and to minimize bleeding-related hypovolemia and anemia through proactive transfusion.

Accordingly, this exploratory study aimed to develop and internally validate clinically prespecified preoperative models for predicting intraoperative red blood cell transfusion in pediatric craniosynostosis surgery and to determine whether the inclusion of fused suture extent improves model performance.

## 2. Materials and Methods

### 2.1. Study Design

This retrospective, single-center, exploratory clinical prediction model development study was conducted in accordance with the Transparent Reporting of a multivariable prediction model for Individual Prognosis Or Diagnosis plus Artificial Intelligence (TRIPOD+AI) guidance [13]. The study was approved by the Institutional Review Board of Kyungpook National University Hospital (approval number: 2026-03-001; 23 March 2026), and the requirement for informed consent was waived because of the retrospective study design. Data access for this study was performed on 23 March 2026.

### 2.2. Participants and Data Source

Patients who underwent craniosynostosis repair surgery between 2014 and February 2026 were included. For patients who underwent more than one craniosynostosis procedure, only the first surgery was analyzed. Patients who underwent concurrent surgery for other craniofacial anomalies were excluded.

All included patients underwent craniosynostosis repair. However, because the aim of this study was to develop a preoperative prediction model, intraoperative variables such as operative duration and anesthesia duration were not considered candidate predictors. No separate external evaluation dataset was available; therefore, the same cohort was used for model development and internal validation.

### 2.3. Outcome and Candiate Predictors

The outcome of interest was any intraoperative red blood cell transfusion (yes/no) during craniosynostosis repair. Outcome information was extracted from the electronic medical records.

Candidate predictors were prespecified on the basis of clinical relevance and prior literature, rather than by univariable screening [14]. The following preoperative variables were considered: age in months [8], weight (kg) [8], American Society of Anesthesiologists Physical Status (ASA-PS) classification [9], preoperative platelet (10^3^/μL) [9], preoperative hemoglobin (g/dL) [3], and the recorded fused cranial suture site, which was the predictor of primary interest in this study. Preoperative hemoglobin and platelet values were obtained within 1 week before surgery.

### 2.4. Preprocessing of Suture Site Information

Because the anatomical distribution of fused sutures was heterogeneous and the sample size was limited, the recorded fused suture pattern was transformed into an ordinal variable representing the extent of fused suture involvement. Cases were classified as single-site for isolated metopic, sagittal, unilateral coronal, or unilateral lambdoid synostosis; double-site for bilateral coronal or bilateral lambdoid synostosis, or for metopic or sagittal synostosis coexisting with unilateral coronal or unilateral lambdoid synostosis; and triple-site for metopic or sagittal synostosis coexisting with bilateral coronal or bilateral lambdoid synostosis. For model development, this variable was entered as an ordinal predictor coded as 1, 2, and 3 for single-, double-, and triple-site involvement, respectively. Fused suture extent was defined as an ordinal measure of the anatomical extent of suture involvement.

### 2.5. Sample Size and Missing Data

No formal sample size calculation was performed because all eligible patients during the study period were included. Given the small cohort and limited number of outcome events, this study was designed as an exploratory, hypothesis-generating prediction model study rather than as a definitive model deployment study [15,16].

Because there were no missing predictor or outcome data in the final analytic cohort, no imputation procedure was required and no patient was excluded on the basis of data completeness.

### 2.6. Model Development and Internal Validation

Because the outcome was binary, the candidate predictor set was low-dimensional and clinically prespecified, we used ridge-penalized logistic regression as the primary modeling approach [17]. Penalization was chosen to reduce overfitting and improve stability in a small-sample, few-events setting [15,16,17]

To evaluate the incremental predictive value of fused suture extent while minimizing data-driven model selection, we prespecified five paired model comparisons (Table A1). In each pair, the model with fused suture extent was designated the full model, and the corresponding model without fused suture extent was designated the baseline model.

No automated predictor selection or univariable screening was performed. All numeric predictors were standardized before model fitting, while the intercept was left unpenalized. The model output was the predicted probability of intraoperative transfusion for each individual patient.

The ridge penalty parameter (lambda) was tuned over a prespecified logarithmic grid (0.001 to 100, 15 values) by inner-stratified five-fold cross-validation, using stratified folds when feasible, and the final value was selected as the lambda yielding the minimum mean cross-validated log-loss [18]. Because random split-sample validation is inefficient in small datasets and may waste information, the cohort was not divided into separate training and test sets [19]. Instead, all observations were used for model development, and internal validation was performed using bootstrap-based optimism correction with 200 bootstrap resamples, with imputation, standardization, and penalty tuning repeated within each bootstrap sample [19]. No class-imbalance correction method was applied because the event fraction was not extremely low and the principal goal was probability prediction rather than forced classification. No clustering analysis was performed because the study used a single-center dataset.

### 2.7. Model Performance Assessment

Model performance was summarized using both apparent and optimism-corrected estimates. The primary performance measure was the area under the receiver operating characteristic curve (AUC) for discrimination [20]. AUC > 0.80 was considered good, values between 0.60 and 0.80 were considered moderate, and values < 0.60 were considered poor [21]. Calibration was assessed descriptively using the calibration intercept and calibration slope [22]. Threshold-dependent classification measures (accuracy, sensitivity, and specificity) were calculated at a probability threshold of 0.5 and were considered secondary descriptive measures, because no clinically prespecified decision threshold was available [23,24].

All analyses were performed using custom code written in MATLAB R2025b (MathWorks, Natick, MA, USA) and SigmaPlot 16 (Systat Software Inc., Palo Alto, CA, USA). Data are presented as mean ± standard deviation for normally distributed continuous variables, median (25th–75th percentile) for non-normally distributed continuous variables, and counts for categorical variables.

## 3. Results

### 3.1. Study Patients

**Figure 1:**
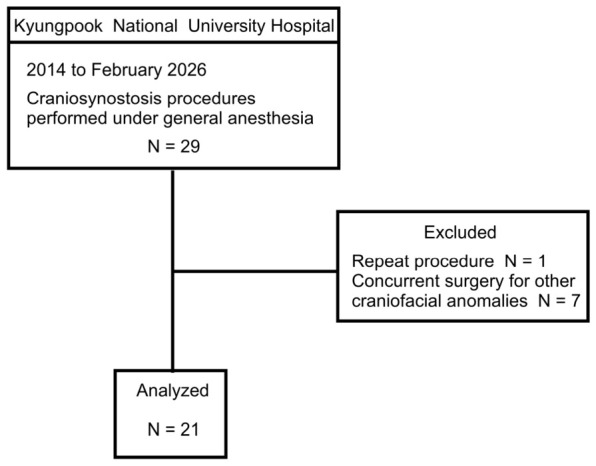
Study flow diagram.

A total of 29 craniosynostosis procedures performed under general anesthesia between 2014 and February 2026 was initially identified. After excluding one repeat procedure in a patient who underwent more than one craniosynostosis operation and seven cases involving concurrent surgery for other craniofacial anomalies, 21 patients were included in the final analysis (Figure 1). A summary of the study cohort is presented in Table 1. No predictor or outcome data were missing among the included patients.

### 3.2. Model Development

Five prespecified baseline/full model pairs were evaluated, yielding a total of eight ridge-penalized logistic regression models. The same 21 patients, including 9 outcome events, were used for model development, hyperparameter tuning, and internal validation because no separate external evaluation dataset was available. Internal validation for all models was performed using bootstrap-based optimism correction, with 200 valid bootstrap replicates obtained for each model.

The lambda values selected by inner-stratified five-fold cross-validation differed across models. For the baseline models, the selected lambda values were 100.0 for Pairs 1, 3, 4, and 5, and 43.9 for Pair 2. For the corresponding full models including fused suture extent, the selected lambda values were 3.728, 1.638, 8.483, 8.48 and 3.728, respectively (Table 2).

### 3.3. Model Specification

The prespecified model pairs are presented in Table A1. The baseline models consisted of weight + preoperative hemoglobin (Pair 1), weight + preoperative hemoglobin + ASA-PS (Pair 2), age + preoperative hemoglobin (Pair 3), age + preoperative hemoglobin + ASA-PS (Pair 4), and age + preoperative hemoglobin + preoperative platelet + ASA-PS (Pair 5). In each pair, the corresponding full model additionally included fused suture extent coded as an ordinal predictor. The model output was the predicted probability of intraoperative transfusion for each individual patient.

### 3.4. Model Performance

**Figure 2:**
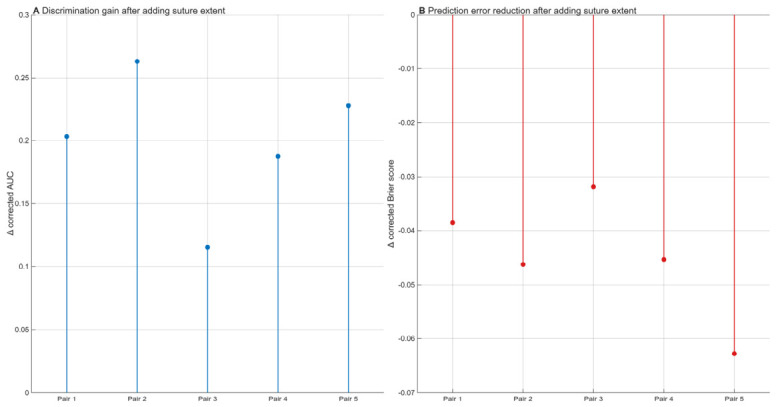
Changes in optimism-corrected model performance after adding fused suture extent. (**A**) Absolute increase in optimism-corrected AUC of the full model compared with the corresponding baseline model across the five prespecified model pairs. (**B**) Absolute change in optimism-corrected Brier score of the full model relative to the corresponding baseline model across the five prespecified model pairs. Negative values indicate lower prediction error in the full model. AUC, area under the receiver operating characteristic curve.

Model performance is summarized in Table 2. Across all five prespecified comparisons, inclusion of fused suture extent was associated with higher optimism-corrected discrimination than in the corresponding baseline model. Corrected AUC increased from 0.470 to 0.674 in Pair 1, from 0.475 to 0.738 in Pair 2, from 0.552 to 0.667 in Pair 3, from 0.516 to 0.704 in Pair 4, and from 0.466 to 0.694 in Pair 5. The corresponding absolute gains in corrected AUC were 0.204, 0.263, 0.115, 0.188 and 0.228, respectively (Figure 2), indicating a consistent incremental enhancement in discrimination across all paired comparisons. Overall prediction error, assessed using the Brier score, was lower in the full models than in the corresponding baseline models.

The best-performing model was the full model including weight, preoperative hemoglobin, ASA-PS, and fused suture extent (Pair 2 full model), which showed an apparent AUC of 0.852, an optimism-corrected AUC of 0.738, and an optimism-corrected Brier score of 0.242.

Calibration intercepts, slopes, apparent accuracies, sensitivities and specificities are summarized in Table A2. The corrected calibration intercepts and slopes varied substantially across the models and deviated considerably from the ideal values of 0 and 1, respectively. These results indicate that calibration was unstable within this small exploratory cohort. In the threshold-dependent descriptive analysis using a probability threshold of 0.5, the baseline models showed apparent sensitivity of 0 and apparent specificity of 1.000, whereas the full models showed apparent sensitivity ranging from 0.444 to 0.556 and apparent specificity of 0.917 across all five models.

**Figure A1:**
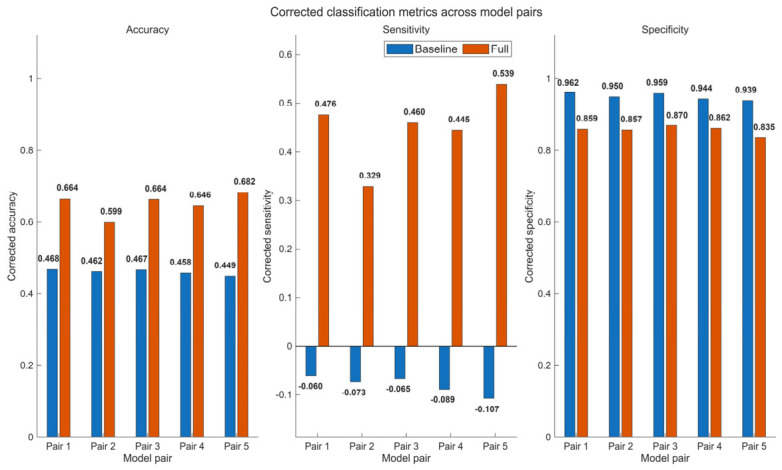
Corrected accuracy, sensitivity and specificity across model pairs.

Optimism-corrected threshold-dependent classification metrics are shown in Figure A1. Across all five prespecified comparisons, the full models showed higher corrected accuracy and sensitivity than the corresponding baseline models, whereas corrected specificity was lower in the full models.

### 3.5. Post Hoc Sensitivity Analysis of Fused Suture Extent Coding

**Figure A2:**
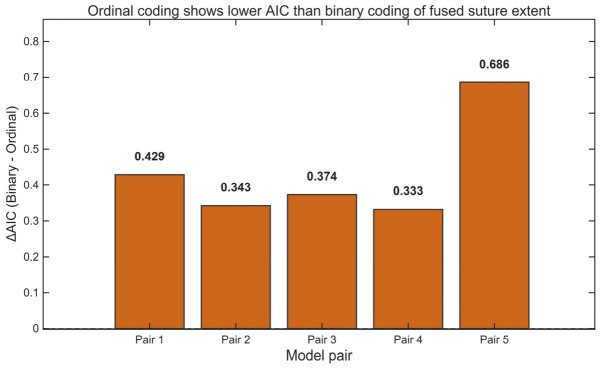
Differences in AIC between binary coding and ordinal coding of fused suture extent, as utilized in the primary analysis, across five model pairs. Positive values favor the ordinal coding approach. AIC, Akaike Information Criterion.

A post hoc sensitivity analysis was conducted to compare the ordinal coding of fused suture extent used in the main analysis (coded as 1–2–3) with a simplified binary coding scheme (single versus multiple). Across all five prespecified model pairs, the ordinal model consistently demonstrated marginally lower Akaike Information Criterion (AIC) values compared to the binary model (see Figure A2). These findings indicate that the ordinal coding approach adopted in the main analysis preserved slightly greater predictive information than the binary classification of single versus multiple in the current dataset.

### 3.6. Model Coefficients

The fitted coefficients of the full models are presented in Table A3. Preoperative hemoglobin showed negative coefficients across all models, whereas fused suture extent showed positive coefficients in all full models. In the age-based models (Full model of Pair 3 and 4), age showed small positive coefficients, and ASA-PS showed negative coefficients in the models in which it was included (Full model of Pair 2, 4 and 5). Weight coefficients were negative in the corresponding full models.

## 4. Discussion

In this exploratory prediction model study, adding fused suture extent consistently improved model performance across all five prespecified comparisons, indicating incremental value beyond conventional preoperative variables in this dataset. Among the prespecified models, the model including weight, preoperative hemoglobin, ASA-PS, and fused suture extent showed the best overall performance, with the highest optimism-corrected AUC (0.738) and the lowest optimism-corrected Brier score (0.242). However, given that the extent of discrimination observed is moderate, the present findings should be regarded as exploratory and intended to generate hypotheses.

Previous transfusion studies in craniosynostosis surgery have largely emphasized perioperative or procedural factors rather than a simple preoperative anatomical descriptor. A recent multicenter retrospective study identified lower preoperative hemoglobin, higher ASA-PS class, lower weight, increasing age, and procedure-related factors as determinants of intraoperative transfusion in patients undergoing craniosynostosis surgery [10]. Although longer operative duration has been associated with transfusion [8,10,25], it is not available before surgery and is therefore less useful for preoperative transfusion risk assessment. In addition, methodologically, previous studies considered only a single suture site when evaluating transfusion risk [10,25]. We therefore focused on preoperative variables and found that fused suture extent improved prediction beyond conventional clinical factors. A recent study that applied supervised machine-learning algorithms on a cohort of 10,732 pediatric craniosynostosis patients from the pediatric database identified prolonged anesthesia duration as the variable most strongly associated with perioperative transfusion [9]. However, since anesthesia duration is an intraoperative variable that is only ascertainable after the patient has entered the operating room, models relying on this variable are not suitable for preoperative transfusion risk stratification. This limitation is particularly relevant given that preoperative risk assessment constituted the primary objective of the present study.

In pediatric spinal surgery, several studies have reported that greater surgical extent is associated with an increased transfusion risk [26,27,28]. Our findings suggest that this relationship may also apply to pediatric craniosynostosis surgery, as patients with a greater number of fused sutures showed a higher likelihood of intraoperative transfusion.

The directions of the model coefficients were mixed (see Table A3). For several variables, the observed directions were consistent with previous literature: higher weight, older age, and higher preoperative hemoglobin were associated with a lower probability of transfusion [8,25]. In contrast, higher ASA-PS was associated with a lower transfusion probability in our study, unlike a previous study in which higher ASA-PS was associated with an increased transfusion risk [8]. This paradoxical finding is likely spurious and probably reflects coefficient instability due to the very small sample size and limited number of events, particularly given that only four patients were classified as ASA-PS III. Nevertheless, despite this limitation, the coefficient for fused suture extent was notably larger than those of other variables in predicting intraoperative transfusion probability, supporting its role as a strong preoperative predictor.

Another notable finding was that adding fused suture extent increased corrected accuracy and sensitivity with a cost of decreased corrected specificity. At the prespecified probability threshold of 0.5, this pattern indicates that the full models more frequently classified patients as being at risk for transfusion, resulting in fewer missed transfusion events but more false-positive classifications. Because sensitivity and specificity are threshold-dependent and strongly influenced by the chosen cutoff, they should be interpreted as secondary descriptive measures rather than the primary basis for evaluating model performance in a prediction model development study [14,20].

Our study suggests that a preoperative model based on routinely available presurgical information may help anesthesiologists identify children who require more proactive blood management planning. In particular, among children undergoing craniosynostosis surgery, the involvement of two or more fused sutures, lower body weight, lower preoperative hemoglobin, and higher ASA-PS may be associated with increased intraoperative transfusion risk, suggesting the need for closer preoperative attention.

This study has several limitations. First, the cohort was small, with only 21 patients and 9 transfusion events. The sample size was insufficient for definitive model deployment, and the present analysis should therefore be regarded as exploratory and hypothesis-generating. Second, the study was conducted at a single center without external validation, so transportability to other institutions remains uncertain. In addition, the calibration results showed substantial instability, with corrected calibration intercepts and slopes deviating markedly from their ideal values of 0 and 1, respectively. These findings indicate that the models may have produced imprecise transfusion risk estimates in this small dataset and further supporting the need for external validation and recalibration prior to clinical application. Third, fused suture extent was simplified into an ordinal variable to preserve statistical stability. Although this approach improved feasibility in a small dataset, it may have obscured heterogeneity among specific suture patterns. Fourth, model performance across potentially relevant subgroups could not be reliably assessed. Accordingly, fairness-related issues, including whether predictive performance differs according to age, sex, ASA-PS, or craniosynostosis pattern, remain unknown and should be examined in larger external datasets.

For implementation, predictor values should be evaluated for completeness, plausibility, and consistency before prediction generation. Body weight and preoperative hemoglobin should be entered using standardized units, whereas ASA-PS and fused suture extent should be assigned according to predefined clinical criteria. If one or more required predictors are unavailable, implausible, or of insufficient quality, the model output should be interpreted cautiously, and a prespecified approach for handling such inputs will be required before clinical application. Because assignment of ASA-PS and fused suture extent involves clinical judgment, the model is not intended to function as a fully automated tool and would require clinician input from users familiar with perioperative assessment in pediatric craniofacial surgery.

Future research should focus on external validation in larger multicenter cohorts with broader clinical heterogeneity and should assess discrimination, calibration, and subgroup performance across institutions. Further studies are also needed to determine whether model updating or recalibration is required in settings with different patient characteristics, surgical practices, or transfusion thresholds. Such efforts will be necessary to establish the applicability, fairness, and generalizability of the model before routine clinical use.

## 5. Conclusions

Fused suture extent improved the preoperative prediction of intraoperative transfusion across all prespecified model comparisons. Our findings suggest that the anatomical extent of craniosynostosis deserves consideration alongside hemoglobin, weight, and ASA-PS when planning perioperative blood management for pediatric craniosynostosis repair. However, because the best-performing model showed only moderate corrected discrimination and developed in a small single-center cohort, the present findings should be considered exploratory. External validation in larger independent datasets is essential before any clinical application.

## Figures and Tables

**Table 1:** Baseline characteristics of the study cohort (N = 21).

Variable	Values
Age (months)	7 (5, 11.5)
Female	11 (52.4)
Weight (kg)	8.7 ± 2.2
ASA-PS I	5 (23.8)
ASA-PS II	12 (57.1)
ASA-PS III	4 (19.1)
Preoperative hemoglobin (g/dL)	12.4 ± 1.1
Preoperative platelet (10^3^/uL)	386 (360.5, 453.0)
Fused suture extent	
Single	15 (71.4)
Double	5 (23.8)
Triple	1 (4.8)
Intraoperative transfusion ^a^	9 (42.9)

Values are presented as number (%), mean ± standard deviation, or median (25th, 75th percentile). ASA-PS, American Society of Anesthesiologists physical status. ^a^ Number of patients who received intraoperative red blood cell transfusion.

**Table 2:** Apparent and optimism-corrected performance of prespecified models with 95% bootstrap confidence intervals.

Pair	Model	Selected λ	Apparent AUC	Corrected AUC	Apparent Brier	Corrected Brier
1	Baseline	100	0.593 (0.481–0.889)	0.470 (0.236–0.686)	0.244 (0.132–0.249)	0.287 (0.242–0.399)
1	Full	3.728	0.769 (0.600–0.973)	0.674 (0.483–0.889)	0.188 (0.055–0.246)	0.248 (0.183–0.379)
2	Baseline	43.94	0.620 (0.537–0.918)	0.475 (0.275–0.648)	0.241 (0.010–0.248)	0.288 (0.240–0.392)
2	Full	1.638	0.852 (0.685–1.000)	0.738 (0.550–0.894)	0.170 (0.001–0.240)	0.242 (0.166–0.378)
3	Baseline	100	0.671 (0.504–0.917)	0.552 (0.325–0.763)	0.243 (0.111–0.249)	0.293 (0.242–0.441)
3	Full	8.483	0.755 (0.596–0.973)	0.667 (0.470–0.888)	0.202 (0.046–0.242)	0.261 (0.198–0.390)
4	Baseline	100	0.657 (0.575–0.973)	0.516 (0.305–0.725)	0.242 (0.071–0.306)	0.306 (0.240–0.452)
4	Full	8.483	0.806 (0.700–1.000)	0.704 (0.510–0.909)	0.194 (0.029–0.261)	0.261 (0.190–0.411)
5	Baseline	100	0.638 (0.583–0.980)	0.466 (0.282–0.657)	0.242 (0.065–0.247)	0.308 (0.241–0.451)
5	Full	3.728	0.824 (0.711–1.000)	0.694 (0.523–0.876)	0.169 (0.001–0.237)	0.246 (0.169–0.374)

AUC, area under the receiver operating characteristic curve; CI, confidence interval. Values before parentheses are point estimates from the original sample, and values in parentheses are 95% bootstrap percentile confidence intervals. Corrected performance estimates were obtained using bootstrap-based optimism correction with 200 bootstrap resamples.

## Data Availability

The datasets generated and analyzed in the current study are available at Zenodo.

## Abbreviations

The following abbreviations are used in this manuscript:

ASA-PS	American Society of Anesthesiologists Physical Status
AUC	Area Under the receiver operating characteristic Curve
TRIPOD+AI	Transparent Reporting of a multivariable prediction model for Individual Prognosis Or Diagnosis plus Artificial Intelligence
AIC	Akaike Information Criterion

## Appendix A. Additional Details of the Preoperative Transfusion Prediction Models

The Appendix provides additional model details, including the prespecified baseline and full model pairs (Table A1), calibration and threshold-dependent descriptive measures (Table A2), corrected accuracy, sensitivity, and specificity across model pairs (Figure A1), a comparison of AIC between binary coding and ordinal coding of fused suture extent adopted in the main analysis across five model pairs (Figure A2), and coefficients of the full models on the original predictor scale (Table A3). The MATLAB analysis code used in this study is available for download from Zenodo (https://doi.org/10.5281/zenodo.19211646).

**Table A1:** Prespcified baseline and full model pairs used for ridge-penalized logistic regression analysis.

Pair 1	Baseline Model	Full Model
1	Weight (kg) + Hb (g/dL)	Weight (kg) + Hb (g/dL) + Fused suture extent
2	Weight (kg) + Hb (g/dL) + ASA-PS	Weight (kg) + Hb (g/dL) + ASA-PS + Fused suture extent
3	Age (month) + Hb (g/dL)	Age (month) + Hb (g/dL) + Fused suture extent
4	Age (month) + Hb (g/dL) + ASA-PS	Age (month) + Hb (g/dL) + ASA-PS + Fused suture extent
5	Age (month) + Hb (g/dL) + PLT (10^3^/uL) + ASA-PS	Age (month) + Hb (g/dL) + PLT (10^3^/uL) + ASA-PS + Fused suture extent

Hb, preoperative hemoglobin; ASA-PS, American Society of Anesthesiologists physical status; PLT, preoperative platelet.

**Table A2:** Calibration and threshold-dependent descriptive measures.

Pair	Model	Corrected Calibration Intercept	Corrected Calibration Slope	Apparent Accuracy	Apparent Sensitivity	Apparent Specificity
1	Baseline	−0.368	6.238	0.571	0.000	1.000
1	Full	−10.786	2.436	0.714	0.444	0.917
2	Baseline	0.491	1.987	0.571	0.000	1.000
2	Full	−7.379	2.447	0.762	0.556	0.917
3	Baseline	3.962	13.927	0.571	0.000	1.000
3	Full	2.335	15.539	0.714	0.444	0.917
4	Baseline	2.648	14.817	0.571	0.000	1.000
4	Full	−1.895	8.431	0.762	0.556	0.917
5	Baseline	3.427	16.603	0.639	0.000	1.000
5	Full	−57.942	−48.585	0.824	0.667	0.917

**Table A3:** Coefficients of the full models on the original predictor scale.

Pair	Term	Coefficient
1	Intercept	0.463
1	Weight (kg)	−0.031
1	Preoperative hemoglobin (g/dL)	−0.154
1	Fused suture extent	1.064
2	Intercept	0.540
2	Weight (kg)	−0.049
2	Preoperative hemoglobin (g/dL)	−0.107
2	ASA-PS	−0.602
2	Fused suture extent	1.572
3	Intercept	0.342
3	Age (months)	0.008
3	Preoperative hemoglobin (g/dL)	−0.128
3	Fused suture extent	0.644
4	Intercept	0.677
4	Age (months)	0.011
4	Preoperative hemoglobin (g/dL)	−0.113
4	ASA-PS	−0.282
4	Fused suture extent	0.648
5	Intercept	−0.398
5	Age (months)	0.019
5	Preoperative hemoglobin (g/dL)	−0.150
5	Preoperative platelet (10^3^/uL)	0.003
5	ASA-PS	−0.399
5	Fused suture extent	1.069

ASA-PS, American Society of Anesthesiologists physical status.
